# Immune mechanisms in arterial hypertension. Recent advances

**DOI:** 10.1007/s00441-020-03409-0

**Published:** 2021-01-04

**Authors:** Ulrich O. Wenzel, Heimo Ehmke, Marlies Bode

**Affiliations:** 1 III. Department of Medicine, University Hospital Hamburg-Eppendorf, Martinistr. 52, 20246 Hamburg, Germany; 2 Department of Cellular and Integrative Physiology, University Hospital Hamburg-Eppendorf, Hamburg, Germany

**Keywords:** Arterial hypertension, End-organ damage, Immunity, Inflammation, Complement

## Abstract

Increasing evidence indicates that hypertension and hypertensive end-organ damage are not only mediated by hemodynamic injury. Inflammation also plays an important role in the pathophysiology and contributes to the deleterious consequences of this disease. Cells of the innate immune system including monocyte/macrophages and dendritic cells can promote blood pressure elevation via effects mostly on kidney and vascular function. Moreover, convincing evidence shows that T and B cells from the adaptive immune system are involved in hypertension and hypertensive end-organ damage. Skin monocyte/macrophages, regulatory T cells, natural killer T cells, and myeloid-derived suppressor cells have been shown to exert blood pressure controlling effects. Sodium intake is undoubtedly indispensable for normal body function but can be detrimental when taken in excess of dietary requirements. Sodium levels also modulate the function of monocyte/macrophages, dendritic cells, and different T cell subsets. Some of these effects are mediated by changes in the microbiome and metabolome that can be found after high salt intake. Modulation of the immune response can reduce severity of blood pressure elevation and hypertensive end-organ damage in several animal models. The purpose of this review is to briefly summarize recent advances in immunity and hypertension as well as hypertensive end-organ damage.

## Introduction

Hypertension remains a leading cause of global death and disability from cardiovascular disease and stroke. High blood pressure afflicts more than 1 billion people worldwide. The borders between normotension and hypertension are arbitrary. In the recent update of the “Guideline for the Prevention, Detection, Evaluation, and Management of High Blood Pressure in Adults,” the threshold for stage 1 hypertension was lowered by 10 to 130 mmHg (Whelton et al. 2017). By reclassifying people formerly considered to have prehypertension as having hypertension, the guideline created a new level of disease affecting people previously deemed healthy. According to this definition, about 46% of US adults have hypertension, as compared with about 32% under the previous definition (Bakris and Sorrentino 2018). It should be noted that in the 2018 European guideline, the threshold was kept at 140 mmHg (Williams et al. 2018).

The working hypotheses for the mechanisms leading to arterial hypertension have been discussed by Coffman (Coffman 2011). The first hypothesis is the “kidney-centric” hypothesis proposed by Guyton. He hypothesized that the kidney was crucial in mediating this relationship between salt and hypertension. He argued that through its functions to regulate volume homeostasis and sodium reabsorption, the kidney could preserve normal blood pressure via pressure natriuresis and that persistent hypertension reflected a failure of the kidney to appropriately excrete sodium. The second hypothesis stresses the critical importance of the control of peripheral vascular resistance. A third hypothesis proposes the skin as an important player by its ability to store sodium and trigger an inflammatory response. A fourth hypothesis proposes that hypertension is an autoimmune disease. Although not mutually exclusive with the others, Guyton’s hypothesis is supported by the largest number of experimental, clinical, and genetic evidence (Coffman 2011). A consensus is emerging from many different vantage points that these four hypotheses share a requirement for some sort of renal damages caused by inflammation, immune infiltration, reactive oxygen species (ROS) activation, or vascular changes (Rossier et al. 2017). Inflammation is indeed found in the kidney, vasculature, and the skin in hypertension.

Two well-described physiological systems that are over activated in hypertension are the sympathetic nervous system and the renin-angiotensin-aldosterone system. Current treatment strategies for hypertension aim to limit the influence of the sympathetic nervous system and renin-angiotensin-aldosterone system on blood pressure, either directly with agents such as adrenoreceptor antagonists (beta blocker, alpha blocker), angiotensin converting enzyme (ACE) inhibitors, angiotensin receptor blockers, and mineralocorticoid receptor antagonists or indirectly using diuretics or vasodilators like calcium channel blockers. However, this approach frequently lacks efficacy, with up to 40% of patients with hypertension failing to achieve adequate blood pressure control, even when prescribed a combination of drugs from three or more classes. These observations highlight that in some patients at least, additional drivers of hypertension must exist and new targets must be defined (Drummond et al. 2019).

A link between hypertension and inflammation has long been suspected (Mattson et al. 2006). Advances in the fields of immunology and mouse genetics from the 1990s onwards brought innovative technologies, more specific research tools and new opportunities to investigate the role of immunity and inflammation in chronic diseases such as hypertension (Drummond et al. 2019). In this review we provide an update of the current knowledge about the role of inflammation in arterial hypertension (Drummond et al. 2019; Norlander et al. 2018; Wenzel et al. 2016) and extend a summary towards what is known about the processes underlying hypertensive end-organ damage. We also discuss a role for high extracellular salt in modulating the differentiation and function of innate and adaptive immune cell populations and provide evidence that salt and immune cell function have effects on the several major players in the pathogenesis of hypertension, i.e., the kidney, the vasculature, and the skin (Wenzel et al. 2019; Wilck et al. 2019).

## Immune cells and hypertension

**Fig. 1:**
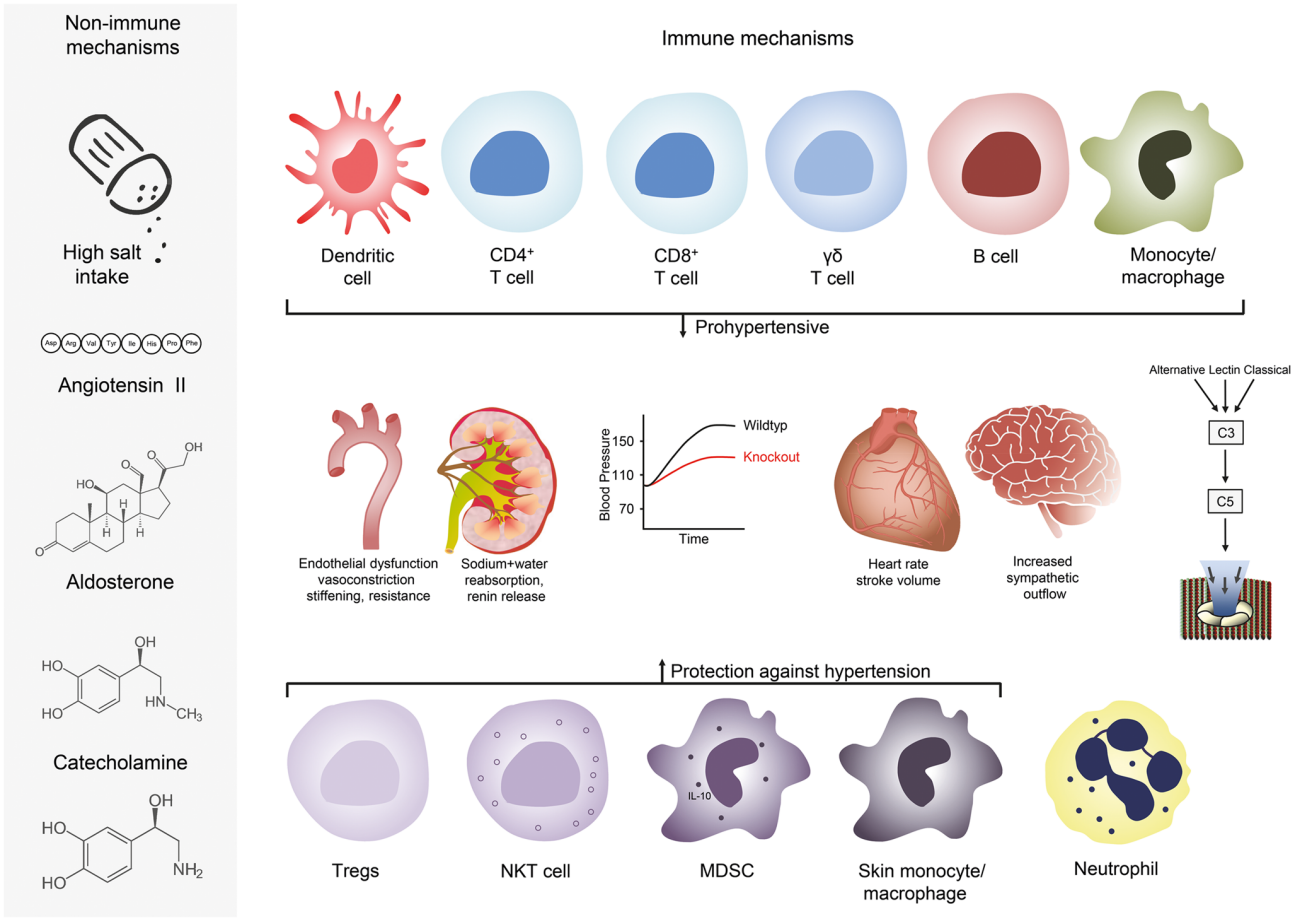
Non-immune and immune mechanisms of hypertension. Non-immune mechanisms like high salt intake, Ang II, aldosterone, and catecholamine increase vascular tone, renal sodium reabsorption, cardiac output, and sympathetic outflow. Immune cells that have been shown to cause or participate in the pathogenesis of hypertension and hypertensive end-organ damage are shown in the upper part. Dendritic cells, CD4^+^ and C8^+^ T cells, γδ T cells, B cells, and monocyte/macrophages promote hypertension. In addition, the complement system also has pro hypertensive properties. Shown in the lower part are immune cells that control the development of hypertension like regulatory T cells (Tregs), natural killer T (NKT) cells, myeloid-derived suppressor cells (MDSC), and skin monocyte/macrophages. The role of neutrophils in the pathogenesis of hypertension is still unclear

To promote hypertension, the immune system must affect the blood pressure-regulating functions of the blood vessels, kidneys, and the heart. A large body of evidence shows that various immune cell subsets infiltrate these organs during hypertension and that the targeted depletion of specific immune cell subsets protects against hypertension in animal models. However, relatively few studies have addressed mechanistic explanations for how immune cells may promote increases in blood pressure (Drummond et al. 2019). In the next sections and summarized in Fig. 1, we give an overview on the immune cell subsets that have been implicated in the development of hypertension and hypertensive end-organ damage and suggest possible mechanistic explanations how they may cause hypertension. Hypertension is clearly caused by classical non-immune mechanisms like high salt intake, angiotensin II (Ang II), aldosterone, and catecholamine. In addition, over the last decade several inflammatory cell types and soluble factors interacting with the vascular system, kidney, heart, and brain have been shown to influence blood pressure levels as shown in Fig. 1 and explained in detail below.

## Innate immune cells in hypertension

### Monocyte/macrophages

Cells of myeloid lineage, including monocytes, macrophages, granulocytes, and dendritic cells, are major effectors of the innate immune system. Selective ablation of lysozyme M-positive myelomonocytic cells markedly blunts angiotensin-induced infiltration of these cells into the vascular wall and attenuates Ang II-induced hypertension and vascular dysfunction (Wenzel et al. 2011). Osteopetrotic (Op/Op) mice, in which macrophages are functionally deficient, exhibit reduced hypertension and vascular remodeling in response to Ang II or DOCA salt (De Ciuceis et al. 2005; Ko et al. 2007). For a more detailed overview of the role of monocyte/macrophages in hypertension and the possible mechanisms, see Fehrenbach and Mattson (2020) and Wenzel (2019).

### Neutrophils

Neutrophils are the most abundant cell type in the human blood and represent an essential part of the innate immune system. Within the Japanese population elevated neutrophil levels were shown to correlate with the risk of developing hypertension (Tatsukawa et al. 2008). The number of neutrophils is increased in the aorta after 7 days of Ang II-induced hypertension (Wenzel et al. 2011). Elevated blood neutrophil activity like myeloperoxidase activity is associated with the height of blood pressure in spontaneously hypertensive rats (Zhang et al. 2018); however, this does not prove causality (Higaki et al. 2019). Adoptive transfer of neutrophils failed to restore the hypertensive response in mice depleted of myeloid cells (Lu et al. 2020; Wenzel et al. 2011). Therefore, direct evidence for a role of neutrophils in hypertension is lacking.

### Reactive oxygen species

It has become clear that reactive oxygen species (ROS) contribute to the development of hypertension via myriad effects. ROS are essential for normal cell function and cells protect the body from invading microbes through ROS generation. However, they mediate pathologic changes in the brain, the kidney, and blood vessels that contribute to the genesis of chronic hypertension. There is also emerging evidence that ROS contribute to immune activation in hypertension. The role of ROS and oxidative stress has been extensively reviewed recently (Loperena and Harrison 2017). The role of ROS in dendritic cells by activating NADPH oxidase is discussed below.

### Dendritic cells

The kidney harbors a dense intricate network of dendritic cells. “Neoantigens” might be produced in response to early hypertensive mechanical trauma injury by induction of cell death and release of intracellular antigens that normally are immune privileged (Wenzel et al. 2016). Interestingly, various hypertensive stimuli increase the production of reactive oxygen species in dendritic cells by activating NADPH oxidase. This leads to lipid oxidation and formation of gamma ketoaldehydes or isoketals. Isoketals rapidly ligate to protein lysines in the dendritic cells forming proteins that are interpreted as non-self. Peptides from these proteins are presented to T cells leading to their proliferation. In addition, isoketal formation also promotes production of cytokines, including IL-1β, IL-6, and IL-23, which polarize T cells to produce effector cytokines. Transfer of isoketal-activated dendritic cells raises blood pressure in wild-type mice but not in RAG-1^−/−^ mice that lack T cells. Moreover, treatment with isoketal scavengers blunts hypertension and its associated renal injury in mice (Kirabo et al. 2014). A role of dendritic cells in hypertension was also suggested by work using abatacept, which blocks CD80 and CD86 on dendritic cells. CD28 binds to these co-stimulatory molecules on dendritic cells and acts as a co-stimulatory receptor in the activation and maturation of T lymphocytes. Ang II and DOCA-salt-induced hypertension were reversed by treatment with abatacept confirming a role of T cell priming by dendritic cells in hypertension (Vinh et al. 2010; Wenzel et al. 2016). Fms-like tyrosine kinase 3 ligand (Flt3L) stimulates the differentiation of migratory and lymph node resident classical dendritic cells. Classical Flt3L-dependent dendritic cells promote renal T cell activation with consequent oxidative stress, fluid retention, and blood pressure elevation after Ang II infusion as shown recently by Lu et al. by using Flt3L deficient mice (Lu et al. 2020).

The most convincing data are coming from the work of Hevia and colleagues (Hevia et al. 2018). Using an approach by depleting of CD11c positive cells using a diphtheria toxin/human diphtheria toxin receptor system, these authors showed that the ablation of myeloid CD11c cells prevents the development of hypertension in response to Ang II infusion with a high-salt diet (Hevia et al. 2018). The salt-sensing serum/glucocorticoid kinase1 (SGK1) in CD11c cells is integral to salt-sensitive hypertension. Mice lacking SGK1 in CD11c^+^ cells have fewer renal inflammatory cells, blunted dendritic cell activation, and less blood pressure elevation in the high-salt/L-NAME model (Lu et al. 2020; Van Beusecum et al. 2019). Thus, dendritic cells are promoting hypertension through effects on T cells in several experimental models (Lu et al. 2020).

### Myeloid-derived suppressor cells

Myeloid-derived suppressor cells (MDSC) are a heterogeneous population of immature myeloid cells that regulate the immune system. They share common features as they all express the myeloid markers, CD11b and Gr1, and the ability to suppress T cell activation. MDSCs are generally considered as one of the several means by which the immune system limits inflammation and thus prevents excessive inflammatory injury (Gabrilovich and Nagaraj 2009). An increased numbers of CD11b^+^Gr1^+^ myeloid cells was found in 3 murine models of experimental hypertension (Ang II, L-NG-nitroarginine methyl ester, and high salt) (Shah et al. 2015). By depleting the MDSCs, blood pressure and renal inflammation were increased and the adoptive transfer of MDSCs to hypertensive mice reduced blood pressure, which demonstrated that the accumulation of MDSCs is a characteristic of experimental models of hypertension and that MDSCs limit inflammation and the increase of blood pressure.

## Adaptive immune cells in hypertension

### CD4^+^ T cells

The role of CD4^+^ T cells and especially T_H_1 and T_H_17 cells has received considerable scrutiny and has been reviewed by us and others (Drummond et al. 2019; Lu et al. 2020; Norlander et al. 2018; Wenzel et al. 2016). The first conclusive evidence for a role of T cells in the pathogenesis of arterial hypertension was provided by Guzik et al. in 2007 by showing that the increase in blood pressure caused by Ang II infusion was significantly blunted in mice lacking the recombinase-activating gene 1 (RAG-1^−/−^ mice), a strain that lacks T and B cells (Guzik et al. 2007). This finding was confirmed in several other laboratories and genetic models (Ji et al. 2014; Madhur et al. 2020; Mattson et al. 2013). Surprisingly, we never observed any Ang II resistance of B6.Rag1^−/−^ mice purchased directly from the Jackson Laboratory as early as 2009 (Seniuk et al. 2020). The Sandberg laboratory reported that Jackson B6.Rag1^−/−^ mice purchased in 2015/2016 lost their resistance to Ang II-induced hypertension (Ji et al. 2017). The reason for these discrepancies is still unclear, but they suggest that yet unidentified no genetic modifiers contribute to the full development of the hypertension-resistant phenotype in B6.Rag1^−/−^ mice in addition to the absence of functional T cells.

Interleukin 17A (IL-17A) and interferon-γ (IFN-γ) released from T_H_17 or T_H_1 cells may stimulate or upregulate transporters and ion channels in the tubular epithelium of the kidney. This includes the sodium hydrogen exchanger 3 (NHE) in the proximal tubule, the Na-K-2Cl-cotransporter (NKCC2) in the thick ascending limb, the sodium chloride cotransporter (NCC) in the distal tubule, and the epithelial sodium channel (ENaC) in the collecting duct. This in turn may cause sodium and volume retention (Kamat et al. 2015; Norlander et al. 2016). As shown for CD11c^+^ cells, SGK1 is an important driver of salt-sensitive hypertension also in T cells (Norlander et al. 2016).

### CD8^+^ T cells

**Fig. 2:**
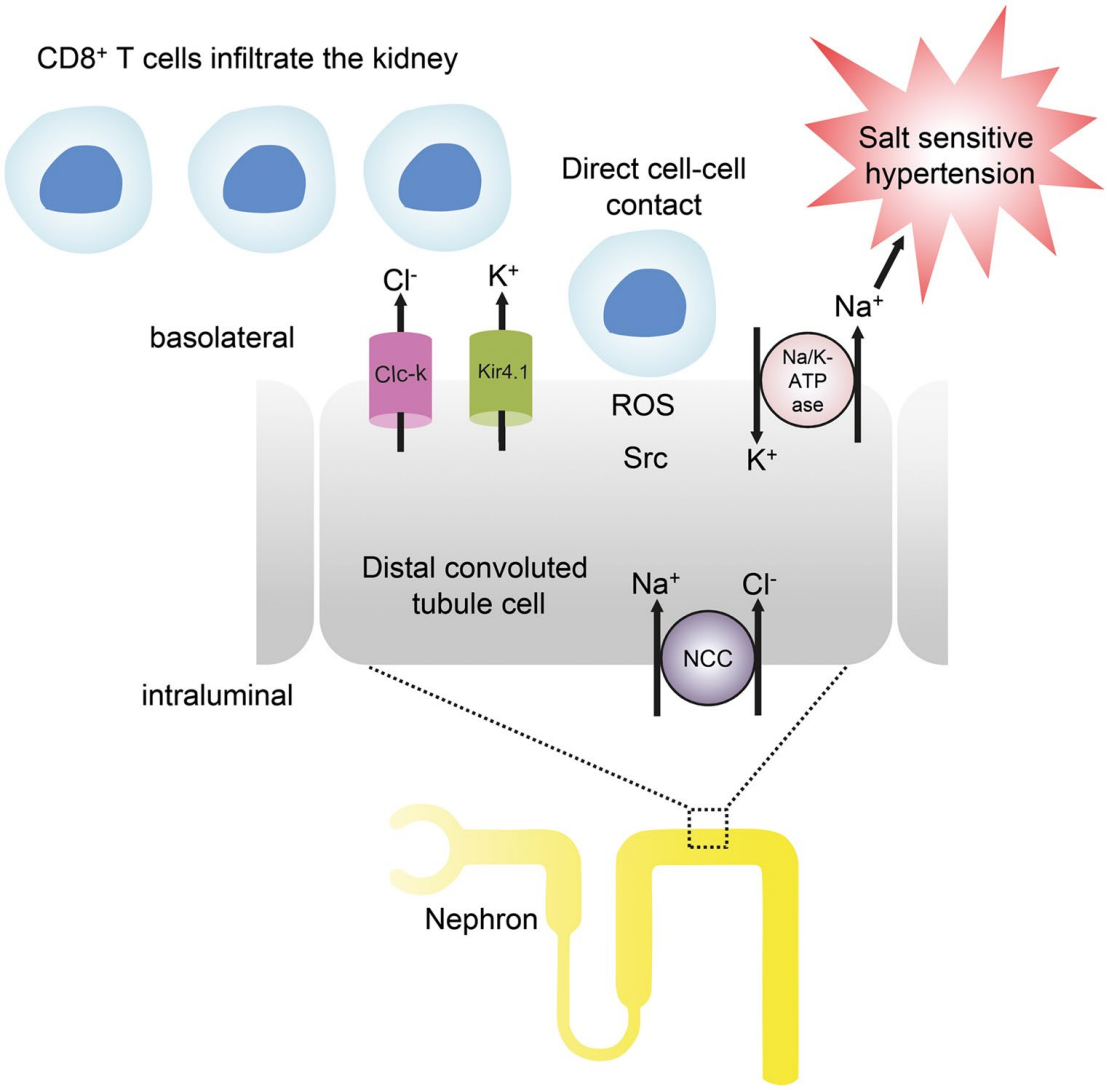
Interaction of T cells and tubular cells. CD8^+^ T cells infiltrate the kidney and directly contact distal tubules cells on the basolateral side. Via ROS and Src signaling activation or upregulation of Kir4.1, ClC-K, NCC, and Na/K-ATPase occurs that causes sodium reabsorption. This sodium retention leads to salt sensitive hypertension ( modified from Wenzel et al. 2019)

While there is convincing evidence that T cells can contribute to the pathogenesis of hypertension, the nature of the underlying mechanisms remains a major open question. CD8^+^ T cells are a major source of IFN-γ and accumulate in the kidney in hypertension. Mice lacking CD8^+^ T cells were found to be protected from hypertension, while mice lacking CD4^+^ cells showed an even increased blood pressure response (Trott et al. 2014). Mice lacking CD4^+^ cells also lack regulatory T cells (Tregs) which may predispose to aggravated hypertension in these animals. Vascular rarefaction in the kidney was noted in wild type and CD4^−/−^ mice, but not in CD8^−/−^ mice. This in turn may cause sodium and volume retention (Trott et al. 2014). Another interesting observation was made by Liu and coworkers and is summarized in Fig. 2) (Liu et al. 2017). In a combined in vivo and in vitro approach, these investigators found that CD8^+^ T cells directly contact the distal convoluted tubule of DOCA-salt mice, thereby inducing an increased expression and phosphorylation of the Na-Cl-cotransporter NCC and the development of salt-sensitive hypertension. The authors showed in co-culture experiments that CD8^+^ T cells induced an upregulation of NCC in tubular cells via an ROS-induced activation of Src kinase. This was leading to an increased efflux of potassium through via the K^+^ channel Kir4.1 and of chloride via the Cl^−^ channel ClC-K, thus causing a compensatory chloride influx via the NCC at the cost of sodium retention. Sodium retention leads to hypertension. Spatial separation of the cells by transwell co-culture prevented these sequence of events, strongly suggesting that a direct physical contact of CD8^+^ T cells and tubular cells is required (Liu et al. 2017).

### γδ T cell

Although T_H_17 cells are considered to be the principal source of IL-17, other cells like γδ T cells have also been reported to produce IL-17. γδ T cells serve as a “first line of defense” or “bridge between innate and adaptive responses.” Genetic knockout of γδ T cells or antibody-induced γδ T cell depletion blunt Ang II-induced blood pressure increases, and endothelial dysfunction suggesting these cells may play a causal role in blood pressure elevation (Caillon et al. 2017; Li et al. 2014).

### B cells

Recent data suggest that also B cells/IgGs are crucial for the development of Ang II-induced hypertension and vessel remodeling in mice. Anti-CD20 therapy protected against Ang II-dependent hypertension as did genetic deficiency of either the B cell activating factor receptor (Tnfrsf13c^−/−^ mice) or the transcription factor Myb (c- mybh/h mice), which renders mice devoid of mature B cells (Chan et al. 2015; Dingwell et al. 2019; Drummond et al. 2019).

### Regulatory T cells

The main function of Tregs is the maintenance of immunological tolerance, i.e., by producing IL-10. Adoptive transfer of Tregs lowers blood pressure and ameliorates cardiac and renal injury in different models of hypertension (reviewed in Wenzel et al. (2016)). Mice deficient in IL-10 develop similar degrees of hypertension but more severe endothelial dysfunction associated with an increased superoxide production during Ang II treatment (Didion et al. 2009). These and other data suggest that Treg-derived IL-10 mediates the beneficial effects of Tregs in hypertension (Kassan et al. 2011). Conversely, IL-2/anti-IL-2 immune complex treatment expands Tregs and reduces aortic stiffening in hypertensive mice (Majeed et al. 2014).

Chen et al. have recently shown in a series of elegant experiments that Ang II-induced hypertension results in an elevated expression of the anaphylatoxin receptors C3aR and C5aR in Tregs. Mice deficient for both receptors had activated Foxp3^+^ Tregs and displayed an attenuated blood pressure response to Ang II as compared with wild type mice. Furthermore, adoptive transfer of C3aR and C5aR double-deficient Tregs showed a more profound protective effect against Ang II-induced blood pressure elevation and renal damage than transfer of wild type Tregs, whereas a depletion of Tregs with CD25 neutralizing antibodies abolished all protective effects (Chen et al. 2018). However, while Chen et al. found that high blood pressure reduced the number of Tregs compared with normotensive mice suggesting that hypertension is a disease with reduced number of Tregs, we observed an increased number of Tregs in the kidney in models of accelerated hypertension (Krebs et al. 2014). Clearly, more work is required to fully understand the mechanisms involved in the blood pressure lowering effects of C5aR1 and C3aR deficiency (Wenzel et al. 2020).

### Natural killer T cells

NKT cells are a unique T lymphocyte sublineage, which is characterized by co-expression of NK receptors and invariant T cell receptors. NKT cells are reactive to the Class I antigen presenting molecule CD1d. CD1d-deficient mice lack NKT cells. In an earlier study, Kirabo et al. observed similar blood pressure changes in response to Ang II in CD1d or Jα18 null mice that are deficient in type I NKT cells compared with wild type mice (Kirabo et al. 2014). In contrast, more recently Wang et al. observed aggravated hypertension and cardiac hypertrophy in Cd1d^−/−^ mice, which was attributed to a reduced production of IL-10. Moreover, administration of the NKT cell activator α- galactosylceramide significantly increased the number of NKT cells and reduced hypertension and cardiac hypertrophy (Wang et al. 2019). Again, further work is needed to clarify these discrepant observations.

### Innate lymphoid cells

NK cells (or “killer” ILC) recognize stressed and damaged cells by a repertoire of activating and inhibitory NK cell receptors. In Ang II-induced hypertension, NK cells migrate into the aortic wall and are a local source for interferon-γ. Depletion of NK cells reduces Ang II-induced vascular dysfunction (Kossmann et al. 2013). The family of “non-killer” ILCs, that have also been referred to as “helper-like” ILC, can be further divided into three groups that differ in their cytokine production, transcription factor usage, tissue localization, and functional characteristic. The role of these cells in hypertension remains undefined.

Despite the overwhelming evidence that inflammation and immunity contribute to hypertension in experimental models, there is a paucity of information to suggest that hypertension or the factors present in the hypertensive milieu lead to T cell activation in humans. Itani et al. recently examined whether also human T cells are activated in hypertension (Itani et al. 2016). They employed a humanized mouse model in which the murine immune system is replaced by the human immune system. Human leukocytes and T cells infiltrated lymph nodes, aorta, and kidney in response to Ang II infusion. They also observed an increase in circulating IL-17A producing CD4^+^ T cells and both CD4^+^ and CD8^+^ T cells that produce IFN-γ in hypertensive compared with normotensive humanized mice. Thus, human T cells become activated and invade critical end-organ tissues in response to hypertension in a humanized mouse model (Itani et al. 2016).

## Complement

The complement system is an ancient part of innate immunity comprising multiple serum proteins and cellular receptors that protect the host from a hostile microbial environment and maintain tissue and cell integrity through the elimination of altered or dead cells. As an important effector arm of innate immunity, it plays also central roles in the regulation of adaptive immunity. Innate and adaptive immune responses have been identified as crucial players in the pathogenesis of arterial hypertension and hypertensive end-organ damage. Thus, complement activation may drive the pathology of hypertension and hypertensive injury through its impact on innate and adaptive immune responses aside from direct effects on the vasculature. The remarkably similar clinical and histopathological features of so-called malignant nephrosclerosis and atypical hemolytic uremic syndrome, which is driven by complement activation, suggest also a role for complement in the development of malignant nephrosclerosis. Indeed, recent experimental data strongly support such a role for complement in all stages of arterial hypertension and hypertensive end-organ damage as reviewed by us and others (Ruan and Gao 2019; Wenzel et al. 2020).

## Salt

The consumption of the so-called Western diet has led to a worldwide increase in sodium intake to levels far above biologically required and recommended limits. These unhealthy eating habits promote hypertension and cardiovascular disease (Wilck et al. 2019). The concentration of sodium in the plasma is relatively constant and maintained at about 140 mmol/l by physiological feedback mechanisms, whereas the sodium concentration in tissues is variable. Traditionally, the kidney was seen as the sole organ that controls body salt content and fluid regulation.

### Salt and skin

**Fig. 3:**
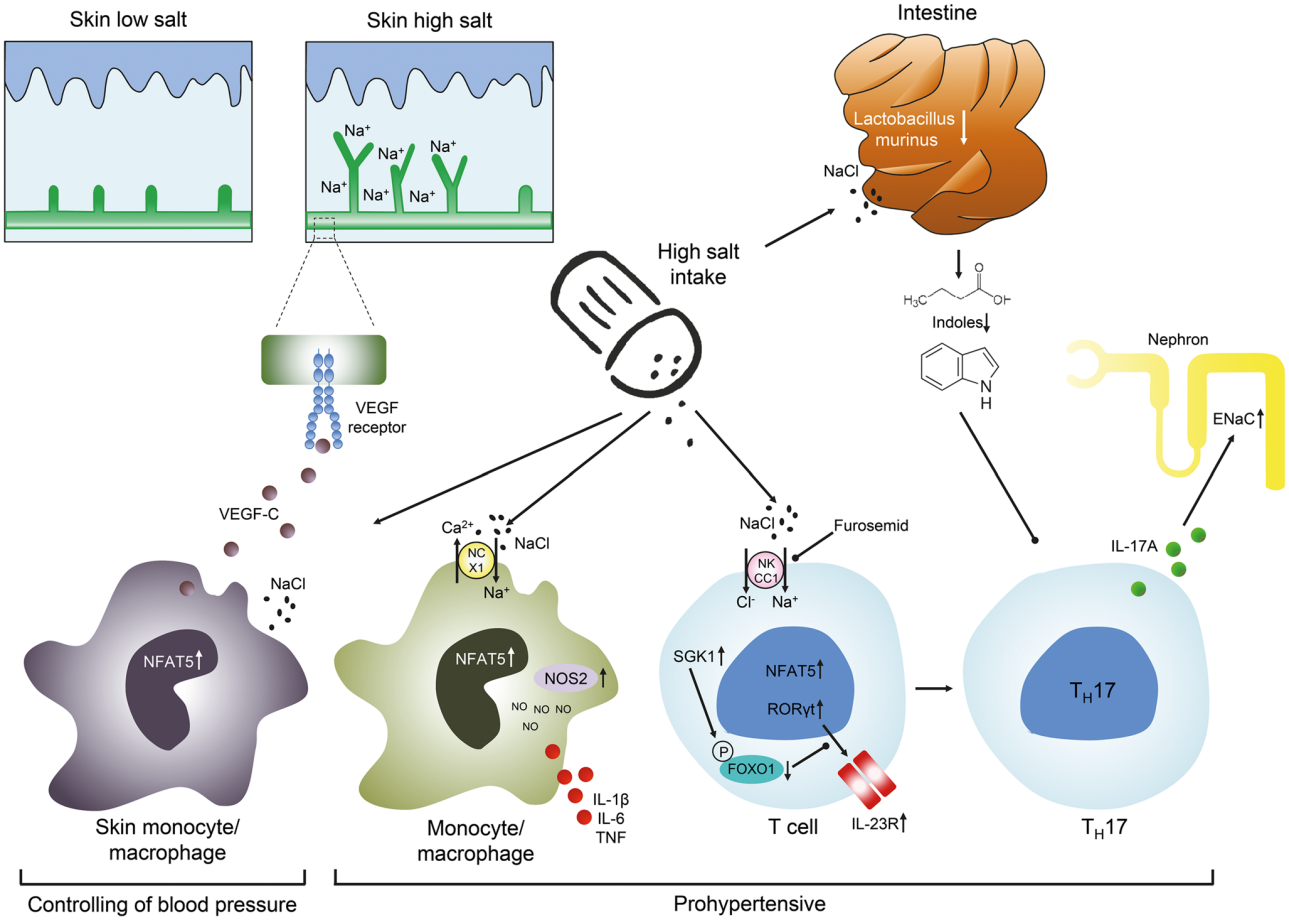
Different effects of high salt intake on inflammatory cells. Increasing sodium intake acting via NFAT upregulates NO synthase in skin monocyte/macrophages and induces the secretion of VEGFc. VEGFc binding to its receptor induces growth of the dermal lymph capillary network that facilitates non-osmotic storage of sodium in the skin and facilitates clearance. This non-osmotic storage of sodium avoids sodium-induced hypertension. High salt enters pro inflammatory monocyte/macrophages via the Na^+^/Ca^2+^ exchanger 1 (NCX1). This triggers NFAT5 expression and subsequent nitric oxide (NO) production by inducible nitric oxide synthase (NOS2). In addition, pro inflammatory cytokines like IL-1β, TNF, and IL-6 are released by these monocyte/macrophages in response to high salt. In T cells, sodium enters the cell by the NKCC1 transporter which can be inhibited by furosemide. Sodium upregulates NFAT5 and its downstream target SGK1. FOXO1 suppresses IL-23R expression which is essential for T_H_17 induction. Phosphorylation of the SGK1 target FOXO1 reduces this suppression enabling RORγt-mediated transcription of IL-23R. In the gut a high salt intake suppresses Lactobacillus murinus and alters the metabolome by decreasing indoles. Decreased indoles result in an increased generation of T_H_17 cells and secretion of IL-17A. IL-17A upregulates the ENaC in collecting duct cells resulting in enhanced sodium reabsorption (modified from Wenzel et al. 2019; Wilck et al. 2019))

Within the last 20 years, however, the interstitium of the skin has emerged as important organ involved in maintaining body sodium balance. For instance, chemical analysis in rodents revealed that the effective osmolyte concentration in skin tissue (i.e., skin (Na^+^ + K ^+^)/skin water) can reach levels of about 190 mM which is substantially higher than the effective osmolyte concentration in plasma of about 145 mM (reviewed in Neubert et al. 2019). The skin interstitium sequesters excess Na^+^ and Cl^–^ in salt-sensitive hypertension as shown in Fig. 3. In rodents fed a high salt diet, sodium accumulates in the skin, creating a local microenvironment that is hypertonic relative to plasma. Much of this sodium appears to be osmotically inactive and bound to negatively charged glycosaminoglycans in the skin. In response to osmotic stress skin monocyte/macrophage upregulate NFAT5 and secrete vascular endothelial growth factor-C (VEGF-C). VEGF-C binds to its receptor on dermal lymphatic vessels and stimulates angiogenesis of dermal lymphatic vessels and lymph capillary hyperplasia that facilitates sodium and chloride clearance from the tissue. Oppositely, failure of such macrophage-driven clearance results in skin electrolyte overload and hypertension. NFAT5 deletion in monocyte/macrophages and VEGF-C receptor blockade induce salt-sensitive hypertension in mice substantiating the important role of monocyte/macrophages located in the skin in blood pressure regulation. Therefore, the non-osmotic skin storage and clearance of sodium avoids sodium induced hypertension (Machnik et al. 2009). Moreover, these experiments demonstrate that monocyte/macrophage signaling exerts homeostatic immune function in the skin by regulating electrolyte clearance, thus contributing to blood pressure control. Corroborating these findings in humans, ^23^Na magnetic resonance imaging shows dermal Na^+^ deposition increases with age and augmented tissue Na^+^ content in patients with refractory hypertension compared with normotensive controls (Kopp et al. 2013). Collectively, these studies constitute a paradigm shift in our understanding of salt and water homeostasis by attributing regulatory functions, previously credited only to the kidney, to larger, more ubiquitous organs like the skin (Wenzel et al. 2019).

### Salt and inflammatory cells

Salt influences cells of the innate and the adaptive immune system. There is robust evidence that increases in Na^+^ levels limit the anti-inflammatory capacity of monocyte/macrophages while promoting their pro inflammatory status as shown in Fig. 3. Sodium enters via the Na^+^/Ca^2+^ exchanger 1 (NCX1) and triggers NFAT5 expression with subsequent nitric oxide production by inducible nitric oxide synthase (NOS2) (Neubert et al. 2020). This induces production and secretion of pro inflammatory cytokines like IL-1β, TNF, and IL-6. In T cells, sodium enters the cell by the NKCC1 transporter which can be inhibited by furosemide. Sodium upregulates NFAT5 and its downstream target SGK1. The transcription factor Forkhead box protein O1 (FOXO1) suppresses IL-23R expression which is essential for T_H_17 induction. Phosphorylation of the SGK1 target FOXO1 reduces this suppression enabling RORγt-mediated transcription of IL-23R, which is necessary for induction of T_H_17 cells. T_H_17 cells secret IL-17A which upregulates the ENaC in collecting duct cells resulting in enhanced sodium reabsorption and hypertension (Wilck et al. 2019; Wenzel et al. 2019). FOXP3^+^ Tregs are central for the maintenance of self-tolerance and can be defective in autoimmunity. Increasing NaCl, either in vitro or in murine models via diet, markedly impairs Treg function (Hernandez et al. 2015). Dendritic cells activated by excess sodium produce increased interleukin-1β (IL-1β) and promote T cell production of cytokines IL-17A and IFN-γ (Barbaro et al. 2017). Sodium enters dendritic cells through amiloride-sensitive channels including the alpha and gamma subunits of the epithelial sodium channel and the sodium hydrogen exchanger 1. Dendritic cells activated by excess sodium produce increased IL-1β and promote T cell production of cytokines IL-17A and IFN-γ. When adoptively transferred into naive mice, these dendritic cells prime hypertension in response to a sub-pressor dose of Ang II (Barbaro et al. 2017).

### Salt and microbiome

The gut microbiome is a new dimension in hypertension research. Wilck and colleagues have recently shown that a high salt intake affects the gut microbiome in mice by depleting Lactobacillus murinus as shown in Fig. 3. The altered microbiome in turn results in an altered metabolome and an increased generation of T_H_17 cells. Depletion of Lactobacillus was accompanied by reduction of the tryptophan metabolites such as indole 3-lactic acid and indole 3-acetic acid. Increased levels of indoles directly inhibit the proliferation of T_H_17 cells in vitro. Treatment of mice with Lactobacillus prevented salt-sensitive hypertension by reducing T_H_17 cells. In line with these findings in mice, a high-salt challenge reduced the intestinal survival of Lactobacillus, increased T_H_17 cells, and increased blood pressure in a pilot study in humans (Wilck et al. 2017). The high salt-induced decrease in Lactobacillus levels in the intestinal microbiome was confirmed by Miranda et al. (Miranda et al. 2018). An increased number of IL-17^+^ lymphocytes in the lamina propria of the small intestine as well as increased levels of IL-17A and the circulation in response to high salt diet in mice was observed by Faracao and colleagues (Faraco et al. 2018). Salt-induced neurovascular changes were ameliorated in IL-17A^−/−^ and lymphocyte-deficient RAG1^−/−^ mice suggesting that dietary salt induces polarization of T_H_17 cells in the small intestine resulting in cardiovascular changes (Faraco et al. 2018).

## Obesity

The prevalence of obesity has increased significantly. Among the many complications of obesity, hypertension is the most common and major one accounting for about 70% in the obese subjects. However, the pathophysiologic factors of obesity‐related hypertension are not well understood at present. Increased circulating levels of leptin, sympathetic overactivity, activation of the renin-angiotensin-aldosterone system, and renal compression participate in obesity caused hypertension. Obesity is also associated with chronic inflammation and a pro-inflammatory phenotype, but the exact role of inflammation causing hypertension in obesity is still not well defined and more work needs to be done (Chrysant 2019).

## Blood pressure measurement

Measurement of blood pressure in mice is challenging. Tail cuff plethysmography in conscious mice is less reliable than in rats. However, with appropriate training and devices, it can be used for screening and large number of mice. Radiotelemetry provides an alternative means of obtaining blood pressure from awake and freely moving mice, without introducing stress artifacts and is the gold standard to measure day and night arterial blood pressure. However, it requires surgery and is costly. In addition, some models of hypertension like DOCA salt involve several surgical procedures like unilateral nephrectomy and implantation of the DOCA pellet. While mice of the C57black strain tolerate well the additional implantation of the telemetric transmitter, in our hands mice of the BALBc strain do not cope well several sequential surgical procedures. A similar observation was recently made by Itani et al. in humanized mice who found that these mice also did not tolerate the two surgeries needed for implantation of telemetry transmitter for blood pressure measurement and subsequent osmotic minipump placement for Ang II infusion, and therefore relied upon tail cuff measurement of blood pressure (Itani et al. 2016). We suggest that tail cuff plethysmography may be used for screening, but that telemetry should be preferred if blood pressure is an important outcome of an experiment if tolerated well by the mouse strain.

## Human data

The importance of the inflammasome in cardiovascular disease is highlighted in the Canakinumab Anti-Inflammatory Thrombosis Outcome Study (CANTOS) (Ridker et al. 2017). This study showed the efficacy of Canakinumab, an anti-IL-1b antibody, at reducing recurrent cardiovascular events in patients with elevated high sensitivity C reactive protein levels. While this study showed no significant change in blood pressure with Canakinumab therapy, an additional analysis was performed that examined the benefit of Canakinumab in groups of participants divided into quartiles based on blood pressure. This analysis revealed a trend of greater reduction in major adverse cardiac events in the highest blood pressure quartile (Rothman et al. 2020). Siedlinski et al. recently identified evidence in support of a potential causal link between elevated lymphocyte count and higher blood pressure in humans. The authors studied the relationship between major white blood cell types and blood pressure in the UK Biobank population and used Mendelian randomization analyses using the UK-Biobank/International Consortium of Blood Pressure-Genome-Wide Association Studies. A positive association between quintiles of lymphocyte, monocyte, and neutrophil counts and increased systolic and diastolic blood pressure was observed. Subsequent testing of lymphocyte count in the context of genetic correlation with renal function or resting and postexercise heart rate demonstrated a positive association of lymphocyte count with urine albumin-to-creatinine ratio (Siedlinski et al. 2020).

## Evolution

Blood pressure control and host defense are such essential mechanisms of homeostasis, and therefore, it is not surprising that evolution incorporated the immune system as active participant in the regulation of blood pressure. Infection can cause hypotension via fluid loss during fever, tachypnea, and diarrhea. Septicemia induces inflammation-related vascular fluid losses. Thus, the risk of hypotension related to inflammation might have favored selection of mechanisms that link immune activation to blood pressure increases for short-term survival benefits. Such an evolutionary force may explain why important antimicrobial effectors could have direct hypertensive effects by promoting vasoconstriction or sodium retention (Wenzel et al. 2016).

## Outlook

Almost 15 years have now passed since the last new class of antihypertensive medications—the renin inhibitors—has entered the market in 2007.

In humans, verification of the experimental evidence that suggests a relationship between salt intake, immune cells, inflammation, and hypertension is a major challenge, but an improved understanding of how salt and immune cells influences in hypertension is crucial and clinically relevant for the identification of therapeutic interventions. Clinical studies which could verify current experimental findings are thus essential but also difficult to undertake. Inflammatory cells and cytokines serve many different functions. Accordingly, their inhibition as a strategy to treat arterial hypertension may result in severe unwanted effects. A better understanding of the exact spatial, temporal, and cellular contributions of the immune system to hypertension, renal, and cardiovascular diseases will be required before novel therapeutic approaches involving immunological targets to combat these disease pathologies may be addressed in drug development programs. Based on what we know today, this will likely require a holistic approach that ideally integrates epidemiological data, large data set analyses about genetic variations in patients, and the functional probing cytokines and immune cells as well as their crosstalk.
